# Supplementary material for: High Electrocaloric Effect in Lead Scandium Tantalate Thin Films with Interdigitated Electrodes
Source: Sensors (Basel). 2022 May 27;22(11):4049. doi: 10.3390/s22114049 (PMC9185452; doi:10.3390/s22114049)
Supplement: Supplementary file 1 [file sensors-22-04049-s001.zip › sensors-1725354-supplementary.pdf]

Supplementary materials

High electrocaloric effect in lead scandium tantalate thin films with interdigitated electrodes

Authors: Veronika Kovacova[1], Sebastjan Glinsek[1], Stephanie Girod[1], Emmanuel Defay[1]

[1]Materials Research and Technology Department, Luxembourg Institute of Science and Technology (LIST), 41 Rue du Brill, L-4422 Belvaux, Luxembourg

1. Summary of samples

Table S1: Table summarizing all PST samples

Number	Substrate	Annealing temperature	Electrode geometry*
1	C-sapphire	900°C	IDE
2	Fused silica	900°C	IDE
3	C-sapphire	750°C	IDE
4	Fused silica	750°C	IDE
5	Platinized silicon	750°C	MIM

*Note that IDE stands for interdigitated and MIM for metal-insulator-metal electrodes.

2. Platinized silicon substrate

Lead scandium tantalate (PST) was also deposited on platinized silicon (Pt(100 nm)/TiOx (20 nm)/SiO2 (400 nm) / (100) Si (675 µm)) (SINTEF) according to the method described in main manuscript. Round-shaped platinum electrodes with 100 µm-diameter were used for MIM geometry on platinized silicon samples (Figure S1 b).

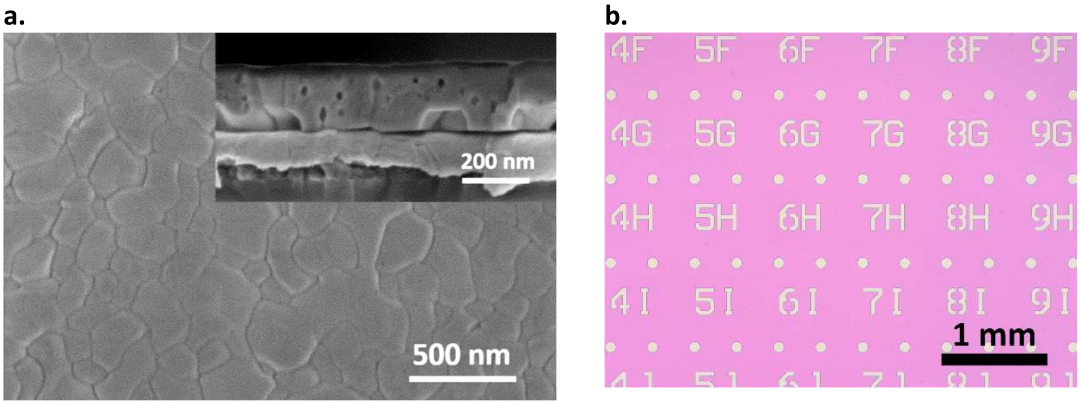

Figure S1: FESEM images of surface and cross-section of a PST thin film on platinized silicon (a) and image of the platinum electrodes on the sample surface (b).

Figure 1.a shows the surface and the cross-section images of PST thin films deposited on platinized silicon. The PST films show a large grain structure (~200 nm and more). In addition, porosity is present in the cross-sectional micrograph.

3. Additional XRD patterns

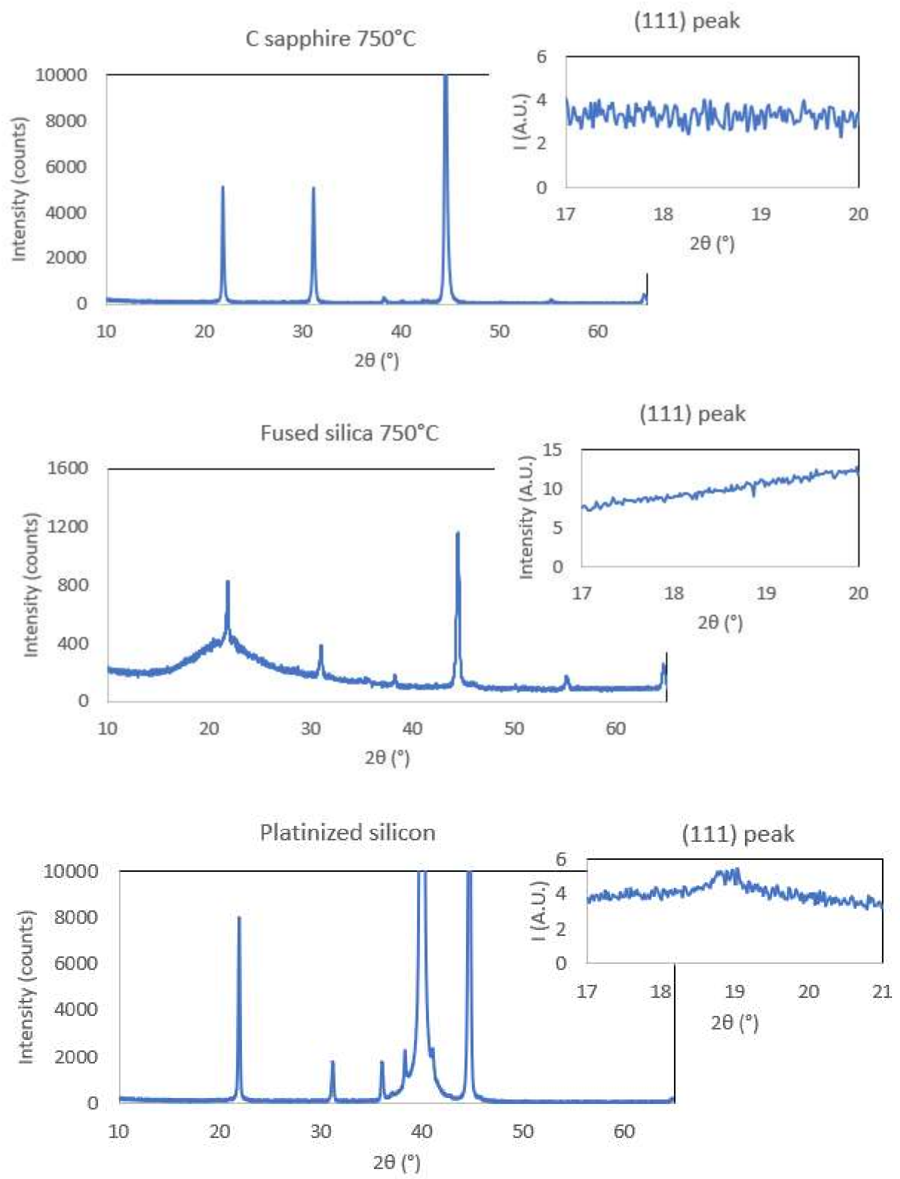

Figure S2: XRD patterns of PST samples on c-sapphire, fused silica and platinized silicon annealed at 750°C with inset images of 111 peak location at χ = 54.7°.

According to the XRD patterns, all PST samples regardless substrate and annealing temperature, are (200) oriented. This preferential orientation is due to the use of the $PbTiO_3$ seed layer.

4. Estimation of the order parameter in PST thin film

All samples are textured and the main texture for all samples is (200) (see Figure S1). In order to observe the maximum intensity for (111) peak, we collect the (111) peak corresponding to the main texture. Therefore, we rotate the sample at psi equal to 54.7°, which is the angle between (200) and (111) planes. In order to determine the order, the (111) superstructure peak needs to be compared to a regular peak. We choose to compare it to the (222) peak, which is at its maximum at the same angle psi equal to 54.7°.

$$S_{111}^{2} = \frac{\left(\frac{I111}{I222}\right) film}{\left(\frac{I111}{I222}\right) ordered}$$

In order to find the ratio between intensities I(111)/I(222) for ordered PST, we referred to the ratio of I(111)/I(200) which is equal to 1.33 [1] and the intensities for random oriented PST from the PDF file number 01-074-2635 [2]. According to the PDF file intensities of (200) and (222) peaks are 64 and 65 respectively. Then I(111)/I(222) is equal to I(111)/I(200) *I(200)/I(222) = 1.33*64/65. I(111)/I(222) for ordered PST equal to 1.309.

The (111) and (222) peaks are further apart than (111) and (200) or (311) and (222) peaks, used for order estimation for random oriented ceramics. For lower 2θ angles, beam spreads over larger surfaces than for higher 2θ angles. In case of thin films, the beam goes through the entire film thickness. Therefore, the diffracting volumes vary as a function of 2θ angle. The peak intensity is directly proportional to the diffracting volume. In order to compare the peak intensities in thin films, diffracting volumes must be taken into account.

Among all PST samples, only PST on platinized silicon showed the presence of (111) peak. The peak was fitted with a pseudo-Voigt function to estimate its area. Similarly, (222) peak was collected and fitted. The order parameter S_{111} was first estimated to be 0.24. However, this value does not take into account the difference of diffracting volumes when collecting (111) peak and (222) peak. The ratio of diffracting volumes for (111) and (222) peaks at χ = 54.7° is equal to 2.0. The integrated areas of (111) and (222) peaks were corrected by the factor from ratio of diffracting volumes and the new order parameter was determined to be 0.17. This value is similar to PST film deposited on platinized silicon annealed at 800°C for 20 min by Brinkman, with order equal to 0.22 [3].

5. Estimation of stress of PST film on fused silica

Calculation of PST stress from thermal expansion coefficient:

$$\sigma = \frac{E_f}{(1-\nu_f)}(\alpha_f - \alpha_s)\Delta T$$

Where σ is stress, E_f is Young's modulus of the film, ν_f is Poisson's ratio of the film, α_f is thermal expansion coefficient of the film and α_s is the thermal expansion coefficient of the substrate, and ΔT is the temperature variation. Taking E_f equal to 1 10^{11} Nm^{-2}, ν_f equal to 0.3, α_f *and* α_s are 6.5 10^{-6} K^{-1} and 0.48 10^{-6} K^{-1} [4] respectively, and ΔT equal to 875 K, the expected tensile stress in PST film on fused silica is 752.5 MPa.

6. Relative permittivity on c-sapphire and fused silica annealed at 750°C

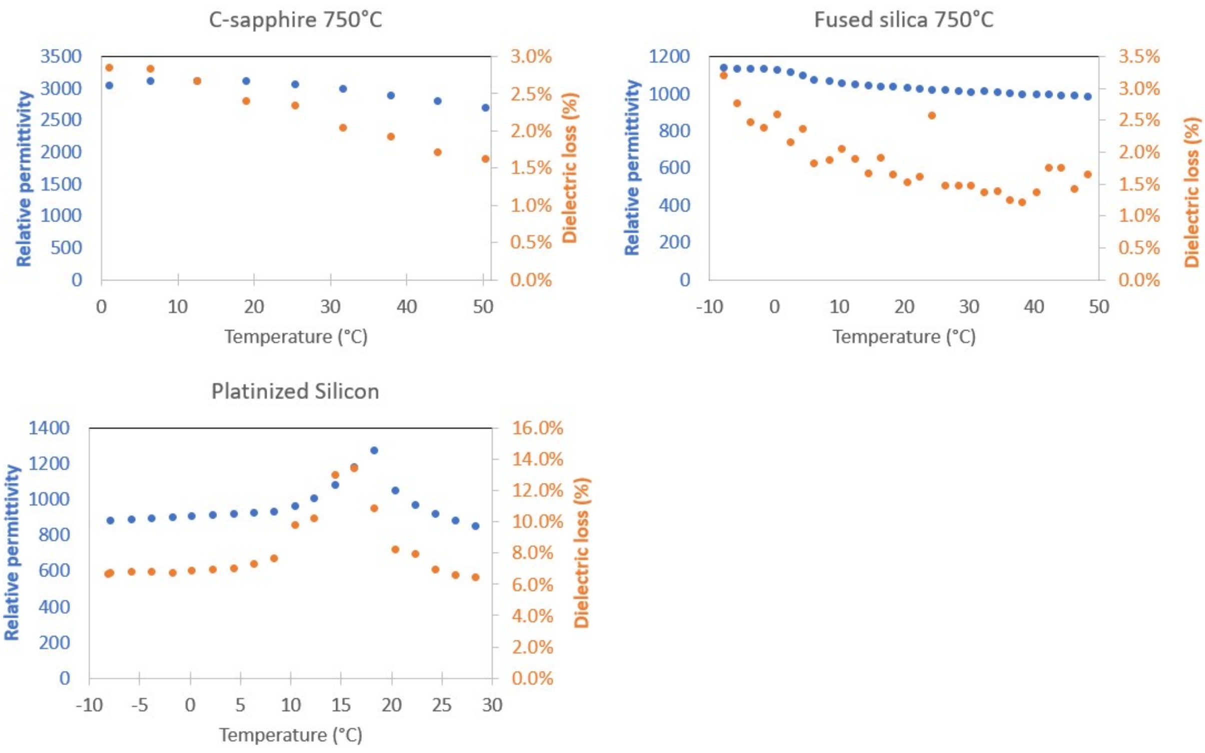

Figure S3: Permittivity versus temperature for PST on c-sapphire, on fused silica and platinized silicon annealed at 750°C. The samples were measured in temperature starting from low to high temperature.

The relative permittivity of PST films deposited on c-sapphire peaks at 3100 for c-sapphire annealed at 750°C. For c-sapphire annealed at 900°C the ε_r reaches 3000. The ε_r on fused silica annealed at 750°C peaks at 1200. This is higher than ε_r on fused silica annealed at 900°C. ε_r decreases with higher annealing temperatures. This difference might be due to denser and thinner films when annealed at higher temperature.

The relative permittivity is equal to 1280 for PST on platinized silicon. This is similar to values obtained on fused silica substrate despite the difference of electrode geometry. It is worth noticing that both fused silica and platinized silicon are inducing large strains into PST layer.

7. Polarization versus electric field versus temperature

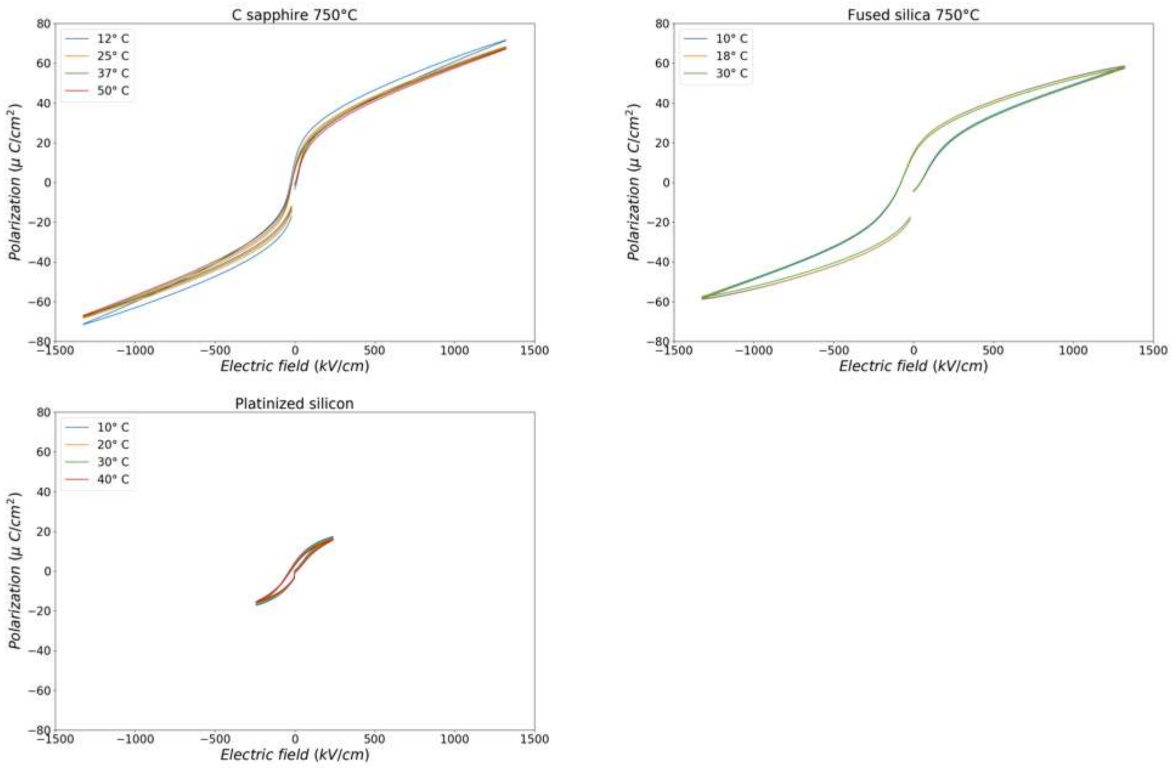

Figure S4: Polarization versus electric field loops for PST on c-sapphire, fused silica and platinized silicon annealed at 750°C.

All samples with IDE geometry were able to withstand 400V corresponding to 1330 kV/cm. Samples on platinized silicon with MIM geometry withstood 4 V at maximum, corresponding to 250 kV/cm.

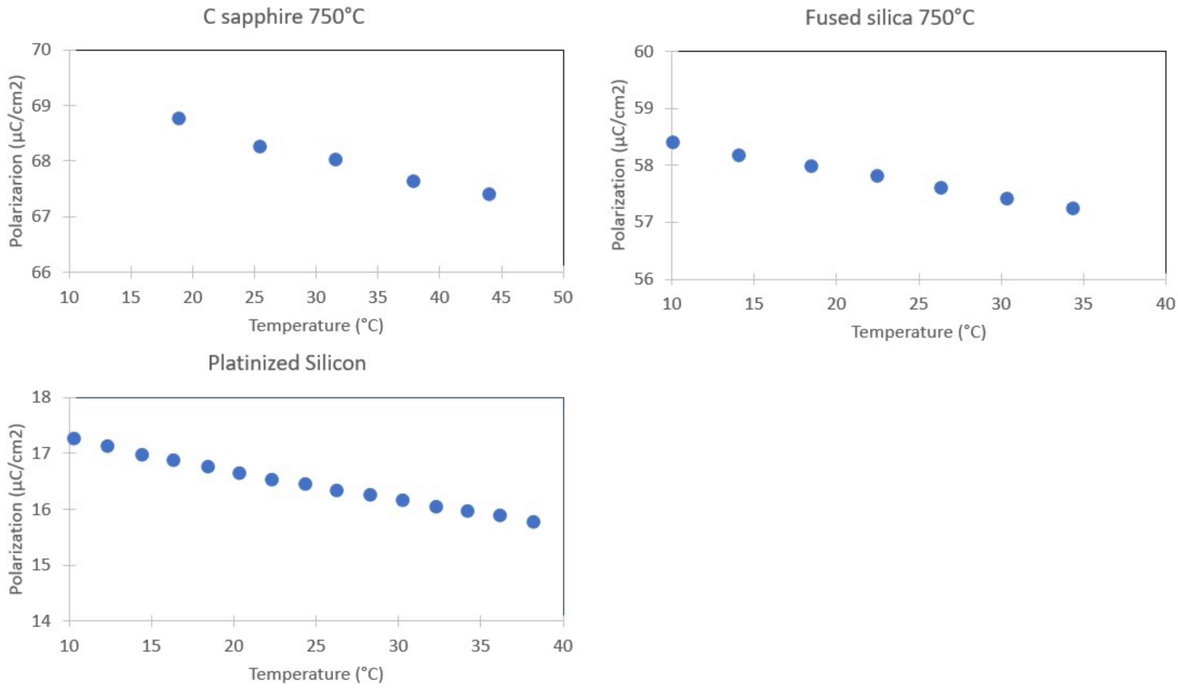

Figure S5: Maximum polarization at 1330 kV/cm versus temperature for PST on c-sapphire and fused silica and at 250 kV/cm for PST on platinized silicon. The polarization versus field was measured with increasing temperature.

8. The electrocaloric effect comparison for all PST samples

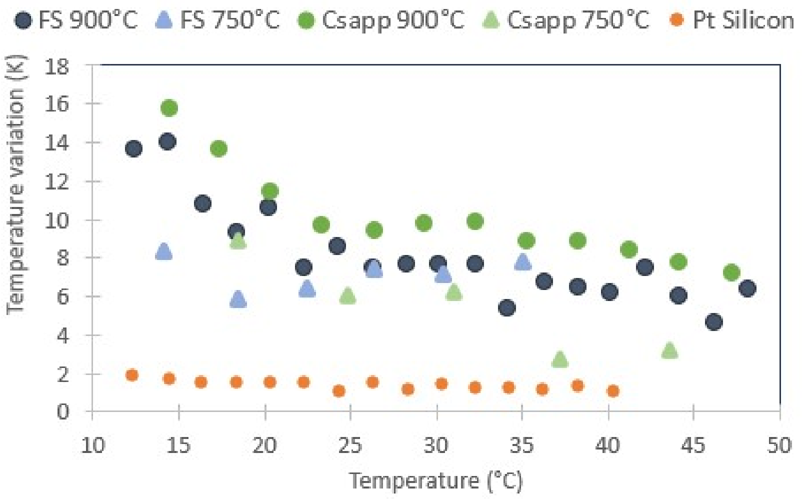

Figure S6: Estimated temperature variation due to the electrocaloric effect versus temperature. PST on fused silica and c-sapphire values correspond to 1330 kV/cm, whereas platinized silica is for 250 kV/cm.

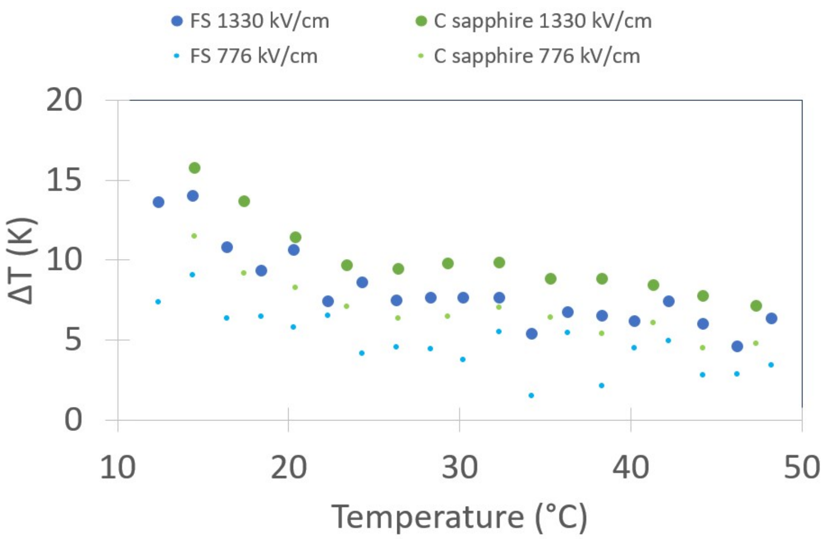

Figure S7: Estimated temperature variation due to the electrocaloric effect versus temperature of PST on fused silica and c-sapphire under 1330 kV/cm and 776 kV/cm.

References:

1. H-Ch. Wang, W.A. Schulze, Order-disorder phenomenon in lead scandium tantalate, J.Am. Ceram. Soc., 1990, 73 (5) 1228-34
2. Dmowski, W., Akbas, M.A., Davies, P.K., Egami, T. "Local structure of Pb (Sc1/2 Ta1/2) O3 and related compounds". J. Phys. Chem. Solids, 2000, 61, 229
3. K. Brinkman, Positional order in lead scandium tantalate (PST) as a tol for the investigation of relaxor ferroelectric behavior in thin films, PhD thesis, EPFL Lausanne Switzerland, 2004
4. Swift glass. Available online : https://www.swiftglass.com/blog/material-month-fused-silica/ (26/04/2022)
